# DLX3 interacts with GCM1 and inhibits its transactivation-stimulating activity in a homeodomain-dependent manner in human trophoblast-derived cells

**DOI:** 10.1038/s41598-017-02120-5

**Published:** 2017-05-17

**Authors:** Sha Li, Mark S. Roberson

**Affiliations:** Department of Biomedical Sciences, College of Veterinary Medicine, Cornell University, Ithaca, NY United States

## Abstract

The placental transcription factors Distal-less 3 (DLX3) and Glial cell missing-1 (GCM1) have been shown to coordinate the specific regulation of *PGF* in human trophoblast cell lines. While both factors independently have a positive effect on PGF gene expression, when combined, DLX3 acts as an antagonist to GCM. Despite this understanding, potential mechanisms accounting for this regulatory interaction remain unexplored. We identify physical and functional interactions between specific domains of DLX3 and GCM1 in human trophoblast-derived cells by performing immunoprecipitation and mammalian one hybrid assays. Studies revealed that DLX3 binding reduced the transcriptional activity of GCM1, providing a mechanistic explanation of their functional antagonism in regulating *PGF* promoter activity. The DLX3 homeodomain (HD) was essential for DLX3-GCM1 interaction, and that the HD together with the DLX3 amino- or carboxyl-terminal domains was required for maximal inhibition of GCM1. Interestingly, a naturally occurring DLX3 mutant that disrupts the carboxyl-terminal domain leading to tricho-dento-osseous syndrome in humans displayed activities indistinguishable from wild type DLX3 in this system. Collectively, our studies demonstrate that DLX3 physically interacts with GCM1 and inhibits its transactivation activity, suggesting that DLX3 and GCM1 may form a complex to functionally regulate placental cell function through modulation of target gene expression.

## Introduction

Establishment of the maternal-fetal interface during human pregnancy is characterized by extensive placental angiogenesis and uterine vascular remodeling to increase placental perfusion to meet the growing metabolic demands of the conceptus^1, 2^. Coordinated with the maturation of placental vasculature, placental trophoblast lineages progressively expand, differentiate, migrate toward and invade the maternal uterus, and thus drive placental morphogenesis^3, 4^. Aberrant placental vasculature has frequently been found to occur coordinately with trophoblast defects in placentae of an array of pregnancy disorders, including the most common and clinically significant complications of preeclampsia (PE) and intrauterine growth restriction (IUGR)^5–8^. A wealth of data supports observations that placental cytokines and transcription factors play important roles in stimulating and modulating trophoblast development, placental angiogenesis, and vascular remodeling across placentation^9–12^; many of which have been found to be dysregulated in PE- and IUGR-complicated pregnancies and have been implicated in disease pathogenesis^13–15^.

A number of placental-specific transcription factors crucial for normal placental development have been described based upon genetically modified mouse models, which in many instances share similar molecular mechanisms with the human placenta^9, 11, 12^. Distal-less 3 (DLX3) is among these factors. DLX3 is a homeodomain-containing protein^16^ that was initially described as a transcription factor involved in epidermal, osteogenic, and hair follicle differentiation during embryonic development^17–20^. Consistent with these findings, naturally occurring mutations of the *DLX3* gene in humans have been associated with ectodermal dysplasia featured by birth defects in hair, teeth and craniofacial bones, as seen in the tricho-dento-osseous syndrome (TDO) and other related developmental diseases^21–24^. Additionally, the potentially causal link between epidermal loss of *DLX3* expression and human skin cancer^25^, and the finding of *DLX3* downregulation in B cells with the MLL-AF4 translocation in acute lymphoblastic leukemia in children^26^ suggest normal postnatal expression of *DLX3* in certain tissue and cell types is also required.

The involvement of DLX3 in murine placental development is well described. *Dlx3*-null mice are embryonic lethal due to placental failure featured by malformation of the labyrinth compartment and an abnormally formed fetal and maternal vascular network^27^. Further, *Dlx3* haploinsufficient mice display disrupted placental vasculature development and altered fetal growth trajectory^28^. Our previous work revealed the *Dlx3*-dependent transcriptome in the mouse placenta and identified multiple pathways in the DLX3-dependent gene regulatory network important for placental development and functional maintenance in human trophoblast-derived cells, including transcriptional regulation of the g*lycoprotein hormone alpha subunit* (*CGA*) a subunit of human chorionic gonadotropin, *matrix metalloproteinase 9* (*MMP9*) and *PGF* ^29–32^. *CGA* is one of two glycoprotein hormone subunits making up human chorionic gonadotropin, a crucial luteotropic signal produced by trophoblasts in the developing conceptus early in pregnancy to signal maternal recognition of pregnancy. Identifying the specific cis-regulatory element within the human *CGA* gene promoter was the initial study linking Dlx3 to the endocrine function of placental trophoblasts. PGF, a proangiogenic cytokine primarily produced by trophoblast cells, plays dual roles in stimulating placental angiogenesis and trophoblast differentiation^33–36^. Moreover, PGF has been implicated to support maternal endothelial system integrity as a free peptide in the maternal circulation^36–39^. Downregulation of placental-derived *PGF* has been shown to be a predictive biomarker and a potential therapeutic target of PE^37–39^. We and others have previously demonstrated that *PGF* expression in human trophoblast-derived cells requires both transcription factors of DLX3 and Glial cell missing (GCM)1^31, 32, 40^. Correlated with this finding, expression patterns of DLX3, GCM1 and PGF overlap remarkably within human placental tissues examined at term; most abundantly in villous cytotrophoblasts (CTB), syncytiotrophoblasts (STB) and extravillous trophoblasts (EVT) in the proximal regions of CTB columns^41–44^. Expression of DLX3 and PGF was additionally observed in endothelial cells surrounding fetal capillaries, highlighting the potential involvement of DLX3 in fetoplacental angiogenesis^41, 43, 44^. Importantly, decreased levels of DLX3 and GCM1 have been reported in placentae complicated with PE, in which *PGF* is dysregulated^45, 46^. Interestingly, our earlier study in human trophoblast-derived cell lines revealed that while DLX3 or GCM1 in isolation were positive regulators of *PGF* gene regulation, their combined impact resulted in a regulatory antagonism in *PGF* expression^32^.

GCM1 is a zinc finger transcription factor with a conserved DNA-binding motif^47^. *GCM1* expression is restricted in the kidney and the thymus in adult mammals and in a subset of trophoblast lineages within the placenta^42, 48, 49^. GCM1 plays a pivotal role in placental development by regulating the expression of several essential genes in trophoblasts cells and is necessary for proper differentiation and functions of trophoblast lineages^50^. For example, GCM1 controls the expression of mouse *syncytins* and the human homolog *ERVFRD-1*, which encode fusogenic proteins which induce CTBs to fuse into multinucleated STBs, and hence the formation of the most essential functional structures of murine and human placentae—the murine labyrinth and human syncytial trophoblast layer^51, 52^. GCM1 also promotes the differentiation and invasiveness of EVTs by upregulating the High-Temperature Requirement Protein A4 to facilitate extracellular matrix remodeling^50, 53^. Notably, decreased GCM1 expression was correlated with defects in both extravillous and villous trophoblast lineages observed in PE-complicated placentae^50, 54^. In mice, *Gcm1* ablation leads to a phenotype similar to that of *Dlx3* null mice, with mid-gestational embryonic death characterized by reduced branching of the chorioallantoic interface and the absence of STB cell lineage leading to placental malformation^55, 56^. Heterozygous *Gcm1* mutants are fertile, but exhibit deficient trophoblast differentiation, abnormal fetoplacental vascularity, and increased syncytial necrosis^57^. Interestingly, wild-type dams carrying *Gcm1* heterozygous fetuses developed late gestational hypertension—one of the hallmarks of PE in humans^57^.

DLX3 and GCM1 share expression domains and functional importance within murine and human placentae. This was highlighted by our previous study where we identified both factors co-localized on the *PGF* promoter^32^. Intriguingly, the combination of DLX3 and GCM1 formed a functional antagonism in regulating *PGF* expression^32^. Based on these observations, we speculated that a physical interaction between DLX3 and GCM1 is likely to functionally coordinate *PGF* regulation. If true, this observation would further our understanding of complex molecular and genetic networks controlling human placental cell function, which may have enormous implications in human pregnancy-associated conditions and numerous adult diseases with origins in early prenatal environment^58, 59^. The goal of the current study was to identify the physical and functional interactions between DLX3 and GCM1 in human trophoblast-derived cells. Our studies provide important mechanistic underpinnings of how a DLX3-GCM1 antagonism influences *PGF* regulation^32^.

## Results

### DLX3 physically interacts with GCM1 and inhibits the transactivation-stimulating activity of GCM1

**Figure 1:**
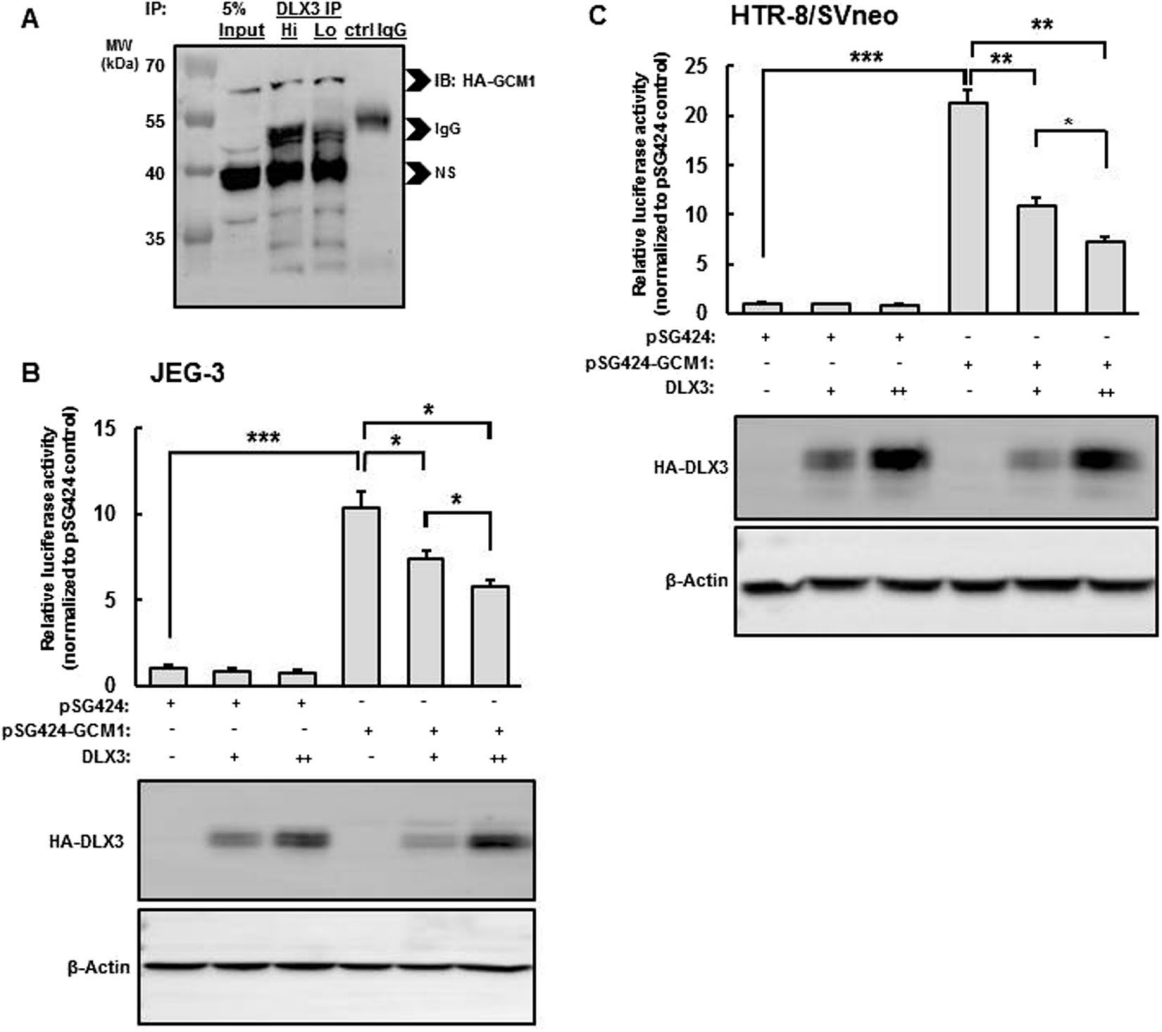
DLX3 physically interacts with GCM1 and inhibits the transcription-stimulating activity of GCM1. (**A**) JEG-3 cells transfected with the pHA-GCM1 for 48 h followed by 10 μM MG-132 treatment for 6 h were collected for immunoprecipitation (IP) using the anti-DLX3 antibody used at two doses (Lo = 1.5 μg/500 μg protein and Hi = 3.0 μg/500 μg protein). IPs were subjected to immunoblotting and probed with the anti-HA antibody. Non-specific rabbit IgG isotype control antibody was used as the negative control for IP. This is a representative complete western blot film from three independent experiments is shown. Molecular mass ladder is shown to the left of the image. NS = non-specific. Mammalian one hybrid assays were performed in JEG-3 (**B**) and HTR-8/SVneo cells (**C**) transfected with pKH3-DLX3 (0.5 µg or 1 µg) and pSG424-GCM1(0.5 µg; Gal4-GCM1) together with pGL4.31(0.5 µg; luciferase reporter) for 48 h and subsequently collected for luciferase assays. In (**B**,**C**) HA-DLX3 protein levels are shown by western blot analysis using β-actin as a lane loading control. The complete blots can be seen in Supplementary Figure 1. Luciferase activity was normalized to protein concentration and to the pSG424 control (empty Gal4 plasmid). Results are presented as the mean ± standard error (SEM) of triplicates from an individual experiment representative of three independent experiments. Asterisks denote a statistically significant difference: *P < 0.05, **P < 0.01, ***P < 0.001 by two-way ANOVA followed by the post-hoc Bonferroni test.

Our previous studies provide strong evidence of colocalization of DLX3 and GCM1 and the functional coordination on the *PGF* promoter in human trophoblast-derived cells^32^; in those studies, both transcription factors were localized by chromatin-immunoprecipitation assay to a novel cis-acting regulatory element on the *PGF* promoter. In the current studies, we hypothesized that DLX3 and GCM1 might physically associate to form a complex to regulate *PGF* expression. Initial pilot studies were carried out to detect such an interaction between endogenous DLX3 and GCM1 in the JEG-3 human choriocarcinoma cell line which constitutively expresses both transcription factors. However, these initial studies were hampered by high relative rates of proteosomal degradation (data not shown). As an alternative, JEG-3 cells were transfected with a GCM1 expression plasmid (pHA-GCM1) expressing the hemagglutinin (HA) epitope-tagged GCM1, and treated with the proteasome inhibitor MG-132 before collection. Cell lysates were subjected to IP using the anti-DLX3 antibody and the IPs were analyzed by immunoblotting with the anti-HA antibody. With this approach, HA-GCM1 was detected in the DLX3 IP, indicating physical interactions between overexpressed HA-GCM1 and endogenous DLX3 (Fig. 1A).

To further confirm the DLX3-GCM1 physical interaction and to test its functional outcome, the GAL4/UAS mammalian one hybrid system was utilized to examine the effect(s) of the DLX3-GCM1 interaction on the isolated transcriptional activity of GCM1 in JEG3 and HTR-8/SVneo cell lines (Fig. 1B,C). HTR-8/SVneo cells are a human EVT cell lineage and express very low levels of endogenous DLX3 and GCM1. This additional cell line was included as a trophoblast lineage reconstitution model to better define the DLX3 and GCM1 interactions. The mammalian one hybrid system is expected to determine the transactivation potential of GCM1 in the absence and presence of DLX3 independently of direct DNA binding of either transcription factor by quantitating the luciferase activity of a reporter gene regulated by Gal4 DNA binding sites. Full length human GCM1 was fused with the DNA-binding domain of the yeast GAL4 (GAL4 DBD) to yield the GAL4 DBD–GCM1 fusion protein. The pGL4.31 reporter vector containing five consensus Upstream Activating Sequences (UAS) for GAL4 DBD (*Gal4*UAS) binding and a minimal adenoviral promoter upstream of the firefly luciferase gene was used to assess the transcriptional activity of GAL4-DBD GCM1 in JEG-3 and HTR-8/SVneo cells overexpressing DLX3. As is shown in Fig. 1B and C, overexpression of GAL4 DBD-GCM1 alone induced significant luciferase activity, correlated with the positive transcription factor nature of GCM1^47^. Consistent with our previous studies^32^, DLX3 overexpression significantly reduced the luciferase activity induced by GAL4-DBD GCM1 in a dose-dependent manner in both JEG-3 and HTR-8/SVneo cells, indicating that the binding of DLX3 with GCM1 in this context inhibited the transactivation-stimulating activity of GCM1. Importantly, overexpression of DLX3 in the absence of Gal4-GCM1 was without effect on this system. Western blot analyses using the HA epitope characterized DLX3 expression levels in this system for JEG3 (Fig. 1B) with similar expression levels in HTR-8SV/neo cells (Fig. 1C). Thus, DLX3 physically interacts with and inhibits the transcriptional activity of GCM1 in human trophoblast-derived cells.

### Construction of DLX3 structural domain expression plasmids

**Figure 2:**
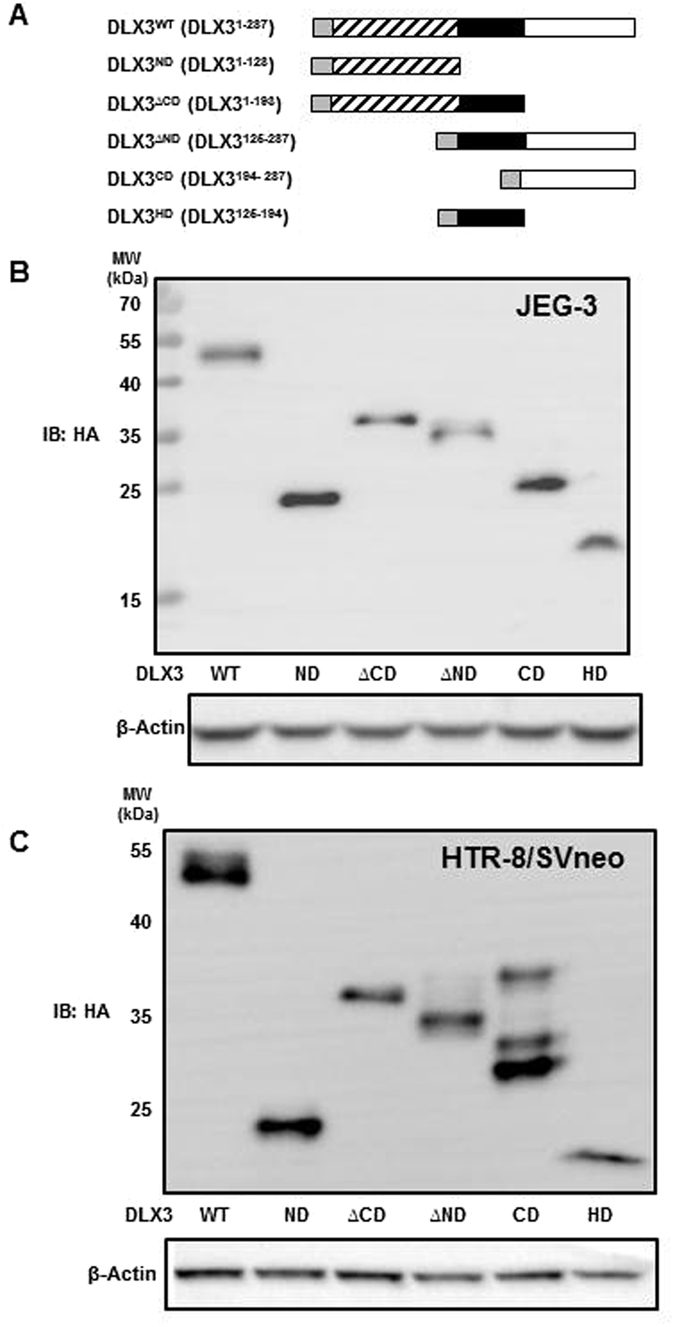
DLX3 structural domains overexpressed as HA-tagged proteins in JEG-3 and HTR8/SVneo cells. (**A**) DLX3 structural domain constructs were clone as schematically illustrated. Grey region: triple HA tag, striped region: DLX3 ND domain, black region: DLX3 HD domain, white region: DLX3 CD domain, ∆: deletion mutant, WT: wild type. Designated DLX3 structural domain expression plasmids were transfected into JEG-3 (**B**) and HTR8/SVneo (**C**) cells for 48 h and then treated with 10 μM MG-132 for 6 h. Cells were harvested for western blot analysis using an anti-HA antibody to examine the expression of HA-tagged DLX3 structural domain mutants. β-actin was also assessed by western blot as an internal loading control. The image depicted is representative of three independent experiments. The blot shown is not cropped.

We next sought to define the region(s) of DLX3 mediating the DLX3-GCM1 physical and functional interactions. The DNA-binding HD and transcriptional activation domains of DLX3 have been described^16^ which formed the framework of our five truncation mutants of DLX3 (Fig. 2A). As a proof-of-concept, these DLX3 structural domain expression plasmids were transfected into JEG-3 cells which were then treated with MG-132 to slow intracellular turnover. Western blots using the anti-HA antibody confirmed that all DLX3 domain mutants were expressed at expected molecular sizes at comparable levels (Fig. 2B). Similar results were obtained in studies using the HTR8/SVneo cell line (Fig. 2C).

### The DLX3 HD is required for the DLX3 and GCM1 physical interaction

**Figure 3:**
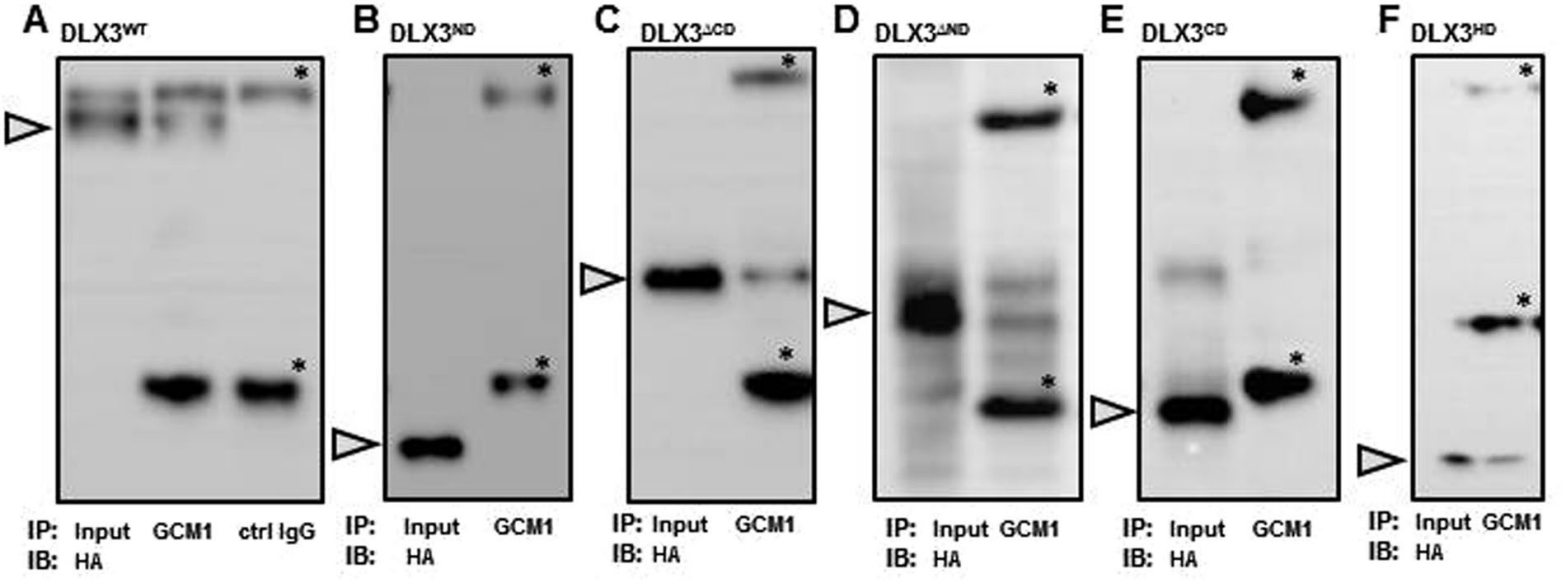
The DLX3 HD is required for a physical interaction between DLX3-GCM1. JEG-3 cells were transfected with indicated DLX3 structural domain expression plasmid for 48 h and treated with 10 μM MG-132 for 6 h. Whole cell lysates were collected for immunoprecipitation (IP) of endogenous GCM1 using the anti-GCM1 antibody followed by immunoblotting with the anti-HA antibody to detect the Dlx3 domain. The panels depict: (**A**) Wild type DLX3^(WT)^; (**B**) Amino-terminal domain of DLX3^(ND)^; (**C**) Deletion of the carboxyl-terminal domain of DLX3^(∆CD)^; (**D**) Deletion of the amino-terminal domain of DLX3^(∆ND)^; (**E**) Carboxyl-terminal domain of DLX3^(CD)^; and (**F**) Homeodomain of DLX3^(HD)^. The grey arrowhead identifies the relevant input bands for the individual domains. Non-specific goat IgG isotype control antibody was used as the negative control for IPs and is shown only for the DLX3WT (**A**; ctrl IgG) but was used in each individual IP (not shown) and heavy and light chain IgGs are denoted with asterisks in each panel. Input reflects 5% whole cell lysates used in each IP. Independent experiments were performed three times and representative cropped images are shown. Uncropped blots are shown in Supplementary Figure 2.

To characterize the DLX3-GCM1 interactions, we first sought to identify regions of the DLX3 protein that mediated the DLX3-GCM1 physical interaction using IP studies. JEG-3 cells transfected with DLX3 structural domain expression plasmids were collected for IP using the anti-GCM1 antibody. IPs were then analyzed by immunoblotting using an anti-HA antibody. Initially, we confirmed the physical interaction between endogenous GCM1 and overexpressed full length HA-DLX3 (DLX3^WT^) (Fig. 3A). Using the similar approach, physical associations were detected between endogenous GCM1 and (i) the carboxyl domain-truncated DLX3 mutant (DLX3^∆CD^), (ii) the amino domain-truncated DLX3 mutant (DLX3^∆ND^) and (iii) the DLX3 HD domain mutant (DLX3^HD^), all of which contain the DLX3 HD domain (Fig. 3C,D,F). In contrast, the DLX3 amino- and carboxyl-terminal domain mutants (DLX3^ND^ and DLX3^CD^) which lack the HD domain were unable to interact with GCM1 in isolation (Fig. 3B,E). These biochemical studies supported the conclusion that the DLX3 HD was essential for the physical interaction between DLX3 and GCM1.

### The DLX3 HD and either the ND or CD are required for full inhibition of the transcriptional activity of GCM1

**Figure 4:**
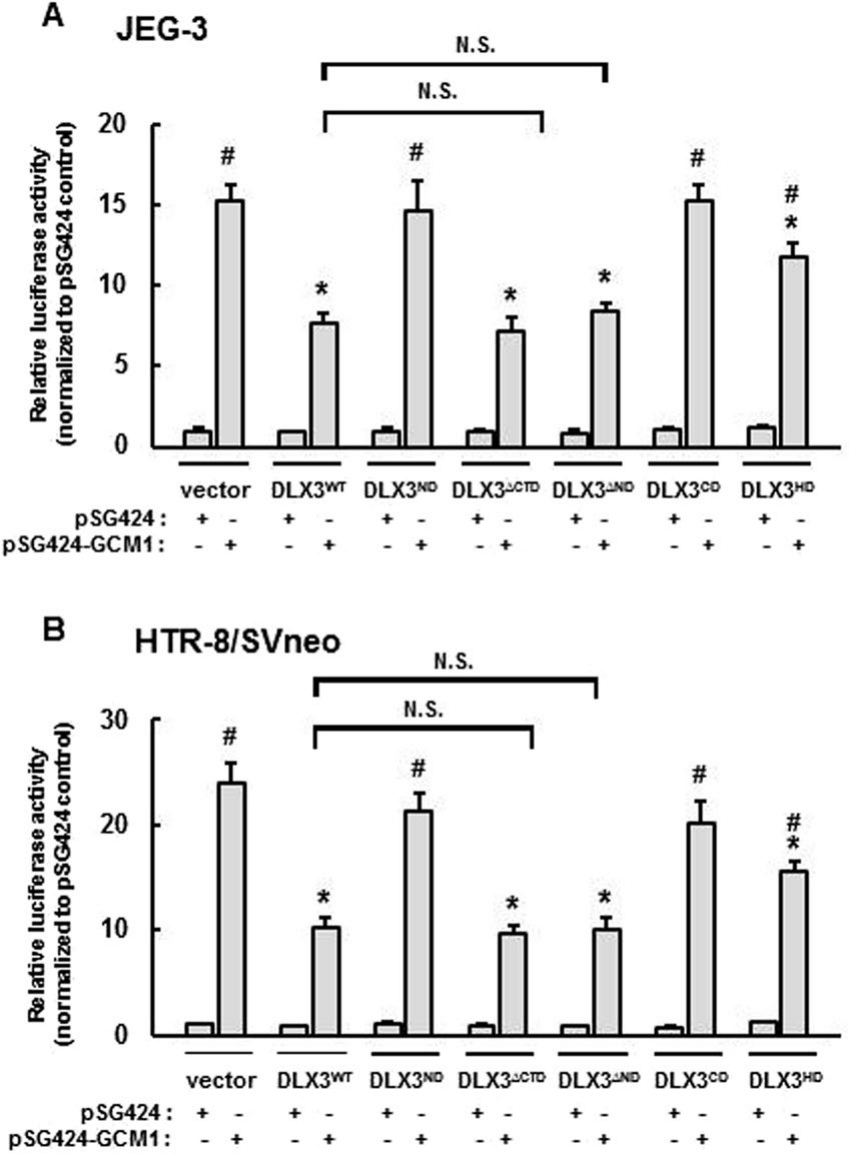
The DLX3 HD and at least one of ND and CD are required for DLX3’s inhibitory effect on the transcription-stimulating activity of GCM1. Mammalian one hybrid assays were carried out in (**A**) JEG-3 cells and (**B**) HTR-8/SVneo cells that were transfected with designated DLX3 structural domain expression plasmid (1 µg) and pSG424-GCM1 (0.5 µg; Gal4-GCM1 fusion) together with pGL4.31 (0.5 µg; luciferase reporter) for 48 h followed by 10 μM MG-132 treatment for 6 h, and subsequently collected for luciferase assays. Luciferase activity is normalized to protein concentration and the pSG424 (empty Gal4 vector) control. Results are reported as mean ± standard error (SEM) of triplicates from an individual representative experiment. Asterisks denote a statistically significant difference compared with the vector control and the # denotes a statistically significant difference compared with the combination of DLX3^WT^ and GCM1 by two-way ANOVA followed by the post-hoc Bonferroni test. N.S. = not significantly different.

In order to assess the importance of these structural DLX3 domains on the functional inhibition of GCM1, we carried out studies using the mammalian one hybrid system in JEG-3 and HTR-8/Svneo cells. Consistent with the biochemical studies described above, overexpression of DLX3^∆ND^ and DLX3^∆CD^ led to significant inhibition in GAL4-DBD GCM1-induced luciferase activity in a manner consistent with DLX3^WT^; whereas DLX3^ND^ and DLX3^CD^ overexpression had no effect on the transactivation activity of GAL4-DBD GCM1 in both JEG-3 and HTR-8/SVneo cells (Fig. 4A,B). Interestingly, the DLX3^HD^ mutant also showed an inhibitory effect on GCM1’s transcriptional activity, but to a less robust extent compared with DLX3^WT^. These results indicate that the DLX3 HD domain is crucial for both physical and functional interactions between DLX3 and GCM1. In addition, the DLX3 HD domain in isolation appears to be insufficient to impart full functional inhibition of DLX3 on GCM1 but required the co-presence of either the DLX3 ND or CD domains, suggesting that the DLX3^ND^ and ^CD^ domains might be important in facilitating or stabilizing the DLX3-GCM1 functional interaction through the homeodomain.

### The DLX3^ΔND^ and ^ΔCD^ contain transactivation-stimulating activities

**Figure 5:**
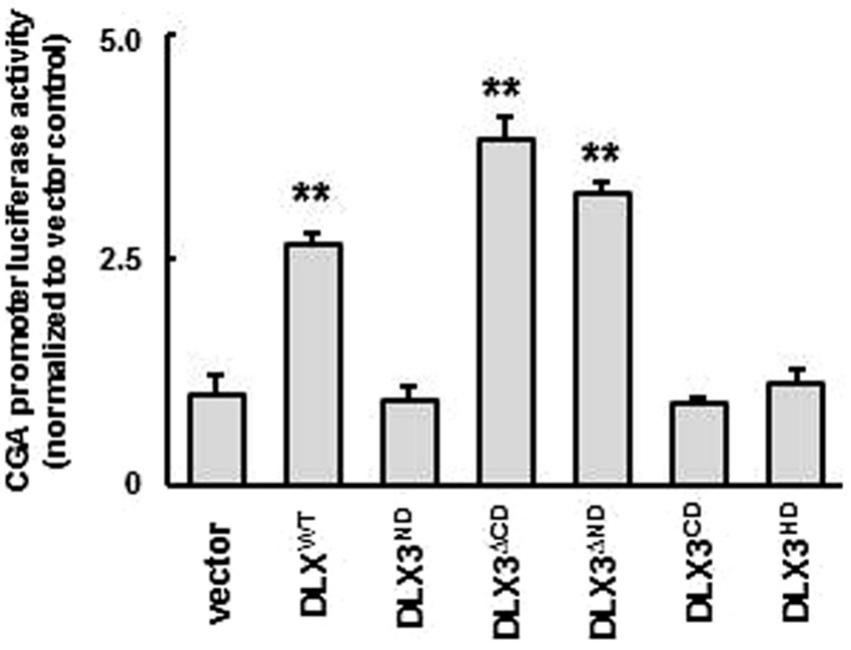
The DLX3 HD and either ND or CD contain transactivation activities. The *CGA* gene promoter has been shown to be transactivated by murine DLX3 and *CGA* gene promoter luciferase reporter assays were performed in JEG-3 cell to assess transactivation activities of regions of the human DLX3 protein. JEG-3 cells were transfected with DLX3 structural domain expression plasmids (1 µg) and *CGA* promoter luciferase reporter plasmid (0.2 µg) for 48 h followed by 10 μM MG-132 treatment for 6 h, and collected for luciferase assays. Luciferase activity is normalized to protein concentration and the vector control. Results are depicted as mean ± standard error (SEM) of triplicates from an individual representative of three independent experiments. Asterisks denote a statistically significant difference compared with the vector control and DLX3^WT^, respectively. *P < 0.05, **P < 0.01 by one-way ANOVA followed by the Student’s *t* test.

Previous studies have described the DNA binding domain, transactivation domains and nuclear localization signal sequence of *Xenopus* and murine DLX3^16, 60^. However, structural studies of human DLX3 and protein-protein interactions have not been described fully. Human DLX3 has 62% identity with *Xenopus* DLX3 and 98% identity with murine DLX3, with 100% identical HD domain amino acid sequence across all three species. Thus we predicted that the human DLX3 might share structural and functional similarities with the murine DLX3. We first sought to test whether the ND and CD both serve as indispensable transactivation domains of human DLX3 using a *CGA* promoter luciferase reporter system, as has been shown for mouse Dlx3 (Fig. 5). *CGA* is a subunit of human chorionic gonadotropin and is a placental gene target of DLX3 in human trophoblast-derived cells^29^. In this study, DLX3 structural mutants were expressed with the *CGA* luciferase reporter in JEG-3 cells. Cells were then collected for quantification of luciferase activity. As anticipated, overexpression of the DLX3^∆CD^ and DLX3^∆ND^ induced significant luciferase activities, whereas DLX3^CD^ and DLX3^ND^ did not possess detectable levels of transcriptional activities, consistent with the requirement of the HD DNA-binding domain in DLX3-dependent gene regulation as a transcription factor (Fig. 5). However, DLX3^HD^ in isolation did not induce any transactivation of the *CGA* promoter, indicating HD alone did not possess any transactivation activities. Interestingly, DLX3^∆CD^ and DLX3^∆ND^ showed consistently increased transcriptional activities compared to DLX3^WT^, suggesting possible autoregulatory mechanisms of the DLX3 transcriptional activity mediated by the CD and ND. These observations suggested that (i) both the DLX3 ND and CD contain transactivation activities of DLX3; (ii) unlike the *Xenopus* and murine Dlx3 where both ND and CD are simultaneously required^16, 60^, for human DLX3, either the ND or CD in conjunction with the HD is sufficient to confer the transactivation activity of DLX3 in the *CGA* reporter system.

**Figure 6:**
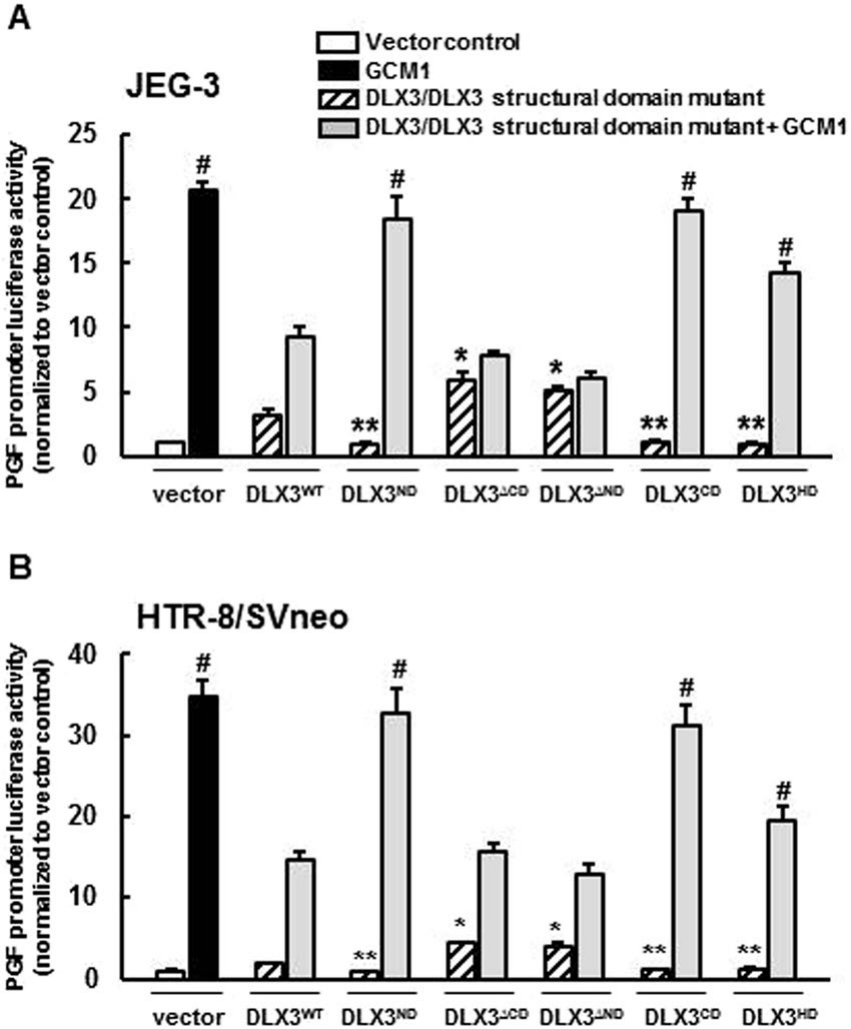
The DLX3 ND and CD facilitate the DLX3-GCM1 functional interaction. The *PGF* promoter luciferase reporter assays were performed in JEG-3 (**A**) and HTR-8/SVneo cells (**B**) to confirm the transactivation activities and GCM1-inhibitory regions of human DLX3. Cells were transfected with designated DLX3 structural domain expression plasmid (1 µg) and pHA-GCM1 (0.5 µg) together with *PGF* promoter luciferase reporter plasmid (0.2 µg) for 48 h followed by 10 μM MG-132 treatment for 6 h, and collected for luciferase assays. Luciferase activity is normalized to protein concentration and the vector control. Results are the mean ± standard error (SEM) of triplicates from an individual representative of three independent experiments. Asterisks denote a statistically significant difference compared with the DLX3^WT^ control, and # denotes a statistically significant difference compared with the combination of DLX3^WT^ and GCM1. *P < 0.05, **P < 0.01, ^#^ *P* < 0.05 by two-way ANOVA followed by the post-hoc Bonferroni test.

To confirm these observations on the *CGA* gene promoter, transactivation activities of DLX3 structural mutants were also assessed in the *PGF* luciferase reporter system. Consistent with the *CGA* luciferase reporter system, overexpression of DLX3^∆CD^ and DLX3^∆ND^ significantly upregulated the *PGF* promoter activity at a level higher than that induced by DLX3^WT^, whereas DLX3^CD^, DLX3^ND^ and DLX3^HD^ didn’t possess any transactivation activities in JEG-3 and HTR-8/SVneo cells (Fig. 6A,B). In these studies, GCM1 were also co-overexpressed with DLX3 structural domains to further assess the functional interactions between DLX3 structural domains and GCM1 on *PGF* regulation. Consistent with observations in the mammalian one hybrid assays, DLX3^ND^ or DLX3^CD^ in isolation showed no effect on the transactivation-stimulating activity of GCM1 on the *PGF* promoter, whereas DLX3^HD^ overexpression alone resulted in a modest but significant antagonism of GCM1 activity (Fig. 6A,B). Again consistent with the mammalian one hybrid system, the co-presence of the HD and either the ND or CD resulted in full antagonism of GCM1 on transactivation of the *PGF* promoter. Taken together, our data revealed that the DLX3 HD and either the ND or CD are required for the functional DLX3-GCM1 interaction regulating the *PGF* promoter activity.

### DLX3 and the DLX3^TDO^ mutant display comparable transcriptional activities and functional inhibition on GCM1

**Figure 7:**
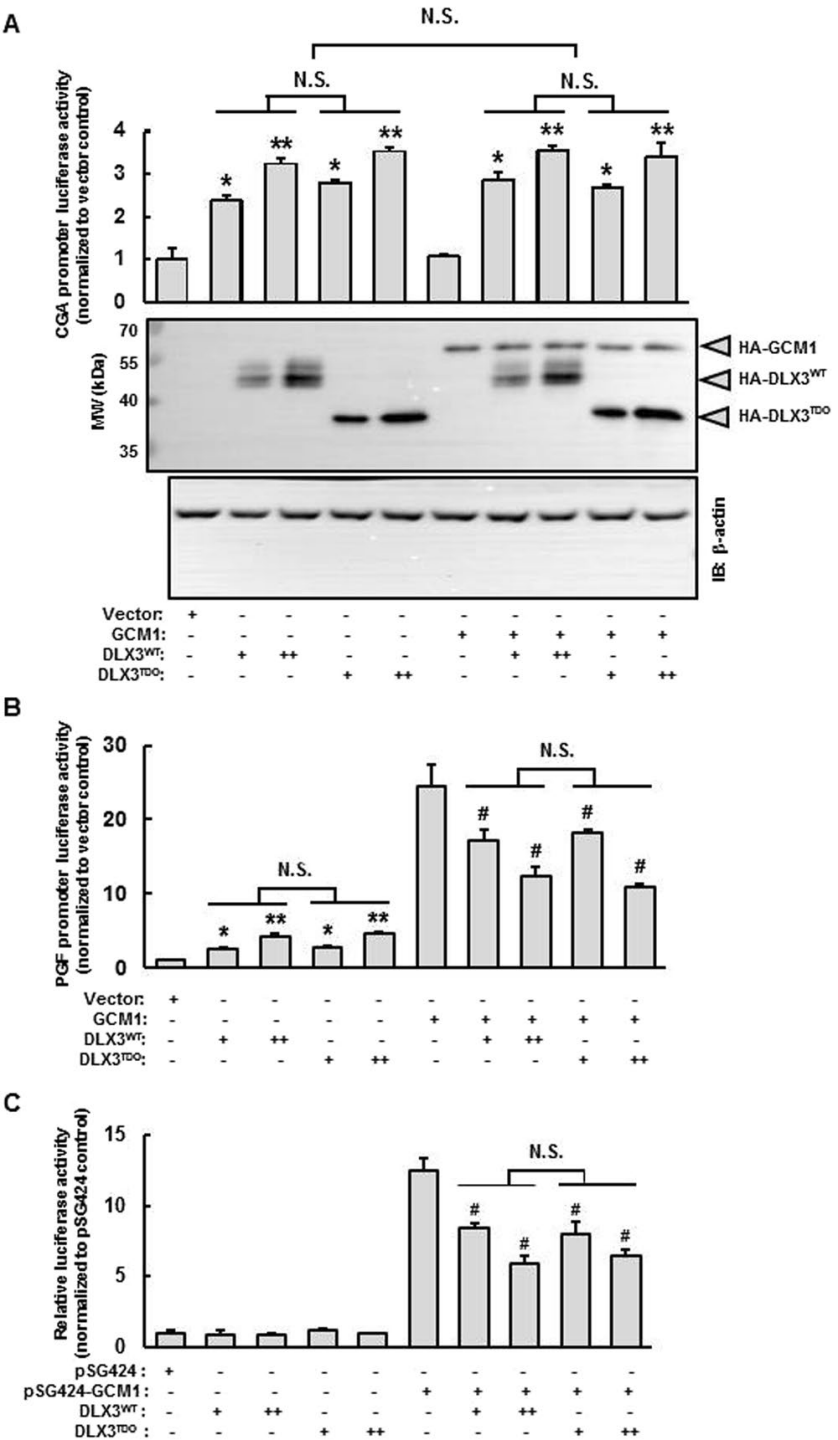
The DLX3 TDO mutant displays similar transcriptional activity and inhibitory effects on GCM1 compared with DLX3^WT^. (**A**) The *CGA* promoter luciferase reporter assays were performed in JEG-3 cells transfected with *CGA* promoter luciferase reporter (0.2 µg), increasing doses of pKH3-DLX3 or pKH3-DLX3^TDO^ (0.5 µg or 1 µg) for 48 h. Western blot analysis was performed to assess expression level of DLX3 variants and GCM1 (see gray arrowheads to the right of (**A**)) using HA immunoreactivity. This is a cropped image. See Supplementary Figure 3 for the uncropped image of this western blot. (**B**) The *PGF* promoter luciferase reporter assays were performed in JEG-3 cells transfected with *PGF* promoter luciferase reporter (0.2 µg) and increasing doses of pKH3-DLX3 or pKH3-DLX3^TDO^ (0.5 µg or 1 µg), without or with pHA-GCM1 (0.5 µg) for 48 h. (**C**) Mammalian one hybrid assays were performed in JEG-3 cells transfected with increasing doses of pKH3-DLX3 or pKH3-DLX3^TDO^ (0.5 µg or 1 µg) and pSG424-GCM1 (0.5 µg) together with pGL4.31 (0.5 µg; luciferase reporter) for 48 h followed by 10 μM MG-132 treatment for 6 h, and collected for luciferase assays. Luciferase activity is normalized to protein concentration and the vector control (**A**,**B**) or the pSG424 control (**C**). Results are reported as mean ± standard error (SEM) of triplicates from an individual representative of three independent experiments. Asterisks denote a statistically significant difference compared with the vector control (**A**,**B**), and # denote a statistically significant difference compared with GCM1 (**B**) or GAL4-GCM1 (pSG424-GCM1) (**C**). *P < 0.05, **P < 0.01, ^#^ *P* < 0.05, N.S.: non-significant differences by two-way ANOVA followed by the post-hoc Bonferroni test.

Naturally occurring mutations in DLX3 have been identified in human populations with TDO syndrome and other ectodermal diseases^21–24^. The classical TDO syndrome is associated with a four-base deletion downstream of the homeodomain in the *DLX3* coding region (c.571_574delGGGG). This mutation is predicted to result in a frameshift in the coding region and thereby a truncated translation product with altered carboxyl terminus^21^. Little is known regarding the activities of DLX3^TDO^ compared to DLX3^WT^ at the molecular level. We speculated that DLX3^TDO^ could potentially behave similar to DLX3^WT^ in our specific promoter analyses since the HD and ND remain intact in the truncated protein. Alternatively, the truncated DLX3^TDO^ protein could be characterized as misfolded due to the truncation leading to an inactive protein. To parse out these alternatives, we assessed the transcriptional activity of DLX3^TDO^ comparing it directly to DLX3^WT^ in regulating placental specific genes in human trophoblast-derived cells by performing the luciferase reporter assays using the *CGA* (Fig. 7A) and *PGF* (Fig. 7B) promoters coupled to luciferase. DLX3^TDO^ and DLX3^WT^ were shown to possess comparable transcriptional activities in upregulating basal expression of *CGA* and *PGF* promoter activities in JEG-3 cells. Importantly, the expression level of the two DLX3 variants could be assessed using the same HA epitope (Fig. 7A) revealing comparable expression levels in this system. As anticipated, the addition of GCM1 with DLX3^WT^ or ^TDO^ did not impact the *CGA* gene promoter. Interestingly, DLX3^TDO^ retained full inhibitory effects on the transactivation-stimulating activity of GCM1 compared with DLX3^WT^ in both the *PGF* luciferase reporter and the mammalian one hybrid assays (Fig. 7B,C). These studies reinforce the specificity of action of DLX3 and GCM1 on *PGF* (compared to the *CGA* reporter) and further suggest that the mechanisms leading to the TDO phenotype in humans are clearly separable from the impact of DLX3 transactivation potential of genes like *CGA* and *PGF* and the functional antagonism between DLX3 and GCM1.

## Discussion

Placental dysfunction leads to pregnancy disorders including gestational diabetes, maternal gestational hypertension, PE and eclampsia, IUGR, abnormal neurodevelopment, preterm birth and stillbirth^61^. Importantly, increasing evidence supports the observation that an adverse prenatal environment in early life linked to a dysfunctional placenta could program the conceptus toward increased risk of a number of chronic diseases developed in its adult life^58, 59^. Thus it is highly desirable to obtain a deeper understanding of the cellular and molecular mechanism underlying placental cell function and maintenance, which would provide invaluable insights into novel preventative and therapeutic strategies with potential for lifelong impact^61^. Here we identified the physical and functional interactions between two important placental transcription factors—DLX3 and GCM1 in human trophoblast-derived cells. Our studies revealed that the DLX3 HD together with either the ND or CD is required for the DLX3-GCM1 interactions. Focusing on DLX3, we further defined the link between structural domains and transcriptional activity of this protein, and demonstrated that both ND and CD retain significant and independent transactivation activities. Our studies implicate the convergence of a DLX3- and GCM1-dependent gene regulatory network at the transcriptional level in human trophoblast-derived cells, which is likely to extend our understanding of molecular mechanisms controlling placental development and functional activities under normal and pathophysiological conditions. The caveat to the present studies is the limitation of trophoblast cell lines and future efforts should include analyses in primary human trophoblast cells and human placentas.

Homeodomain-containing proteins fulfill a wide spectrum of biological functions as regulators of gene expression throughout embryonic development and postnatally in axon guidance and tissue patterning, migration, regulation of plasticity and differentiation^62–64^. We and others have proposed a role of HD proteins in placental vascular development, represented by the HD family member DLX3, as targeted deletion of *Dlx3* results in mid-gestation fetal death due to placental vascularization abnormalities in mice^27^. In our earlier effort to define gene targets and molecular mechanism underlying the regulatory activities of DLX3, we identified *PGF* as one of the direct gene targets of DLX3 in the mouse placenta and human trophoblast-derived cells, and therefore proposed a mechanism by which DLX3 engages in placental angiogenesis and trophoblast differentiation and survival, where PGF plays a pivotal role^31, 32^. In the present study, we provide further evidence for a novel role of DLX3 as the inhibitory modulator of transcriptional activity of an important determinant of placental function, GCM1. While our studies using the *in vitro* human trophoblast cell model have clear limitations, findings reported here may have important implications in the *in vivo* physiological conditions within the human placenta. DLX3 has been previously showed to be a modest regulator of *PGF* compared with GCM1, which strongly induces *PGF* gene transcription^32^. While speculative, by binding with GCM1 and inhibiting the transactivation-stimulating activity in a dose-dependent manner, DLX3 may serve as a “molecular brake” to fine-tune GCM1-dominated *PGF* expression, and therefore potentially control placental vascular expansion and trophoblast overgrowth.

Our studies revealed the central role of the DLX3 HD domain in mediating the DLX3-GCM1 physical and functional interactions. This is not surprising considering the HD domain also mediates the DLX3-MsxI and DLX3-Smad6 interactions^60, 65^. However, unlike in the latter cases where the DLX3-MsxI and DLX3-Smad6 associations interfere with the binding of the DLX3 HD to DNA and thus lead to loss of DLX3’s transcriptional activity^60, 65^, the DLX3-GCM1 interactions appear to have no effect on DLX3’s transcriptional activity^32^. Conversely, we speculate that the interaction with GCM1 might facilitate the association and binding specificity of DLX3 on the *PGF* promoter. The HD domain is known for promiscuous DNA binding with a general preference for motifs centered with the TAAT sequence^62, 63, 66^. Many HD proteins act in conjunctions with their binding partners to impart their DNA binding affinity and specificity^63, 67^. It is likely that interactions between DLX3 and its binding partners including GCM1 serve as a means to distinguish specific gene targets from other TAAT-rich sequences in the genome, depending on cellular contexts and physiological states. This is supported by the lack of DLX3 consensus binding sites on the *PGF* promoter region where both DLX3 and GCM1 localize in the proximal portion of the *PGF* promoter^32^. Notably, different binding partners would lead to variable regulatory outcomes in combination with DLX3. For example, DLX3 and ETS2 have been reported to synergistically transactivate the *bovine Interferon-Tau* and the CGA promoters in human trophoblast-derived cell lines *in vitro* ^68, 69^; and here we demonstrate that DLX3 and GCM1 antagonistically regulate *PGF* expression in human trophoblast-derived cells. Thus, we postulate that the DLX3-GCM1 interactions might affect specific functional activities of both factors from different aspects: while DLX3 inhibits GCM1’s transcriptional activity, GCM1 might facilitate the specificity of DLX3 binding on the *PGF* promoter.

The DLX3 ND and CD domains appeared to be important for full inhibition of DLX3 on GCM1’s activity, suggesting that both domains might be involved in the DLX3-GCM1 interactions, and possibly mediate interactions between DLX3 and other binding partners. This is consistent with previous findings in Homeobox (HOX) genes—a subfamily of HD proteins, where regions outside of the HD domain could play multiple roles including regulating interactions between HOX and specific binding partners, providing additional specificity to gene targets of HOX, and helping to interpret input through signaling cascades^70^. In addition, our study here indicated that the transactivation domains of human DLX3 reside in both ND and CD domains, consistent with the findings in the murine and *Xenopus*. However, unlike the murine and *Xenopus* Dlx3 which require the presence of both the ND and CD to induce Dlx3-dependent transactivation, we showed that either one of the human DLX3 ND or CD in conjunction with the HD DNA binding activity was sufficient to confer DLX3’s transactivation activity in upregulating promoter activities of *CGA* and *PGF*. Intriguingly, deletion of either the ND or CD resulted in increased transcriptional activities of DLX3 mutants, possibly due to potential conformation changes in the folding of the truncated proteins, or loss of post-translational modification sites conferring transactivation inhibition of DLX3. The DLX3^TDO^ mutant included in our study displayed similar transcriptional activity and functional interactions with GCM1 compared with wild-type DLX3, suggesting the DLX3 CD domain is dispensable for DLX3-dependent upregulation of *CGA* and *PGF*, and further is potentially dispensable for DLX3’s functional activities in the placenta. It is presently unclear if pregnancy disorders associated with malformation of the placenta occurs in the TDO-affected population. Our study suggests that the DLX3 CD is likely to be involved in tissue-specific signal interpretation that contribute to highly precise biological functions of DLX3 in different tissues and cellular contexts.

In conclusion, we have uncovered novel roles of DLX3 as a binding partner of GCM1 and a negative regulator of GCM1’s transactivation-stimulating activity in human trophoblast-derived cells, thus providing a mechanistic explanation of the antagonistic regulatory effect on *PGF* regulation by the combination of DLX3 and GCM1. The DLX3 HD domain mediates the physical and functional interactions between DLX3 and GCM1, whereas the ND and CD domains appeared to further facilitate the interactions. We characterized that both the DLX3 ND and CD conferred the transactivation activity of DLX3, correlated with their potential involvement in cofactor binding and signaling interpretation. Our study helps to define the inter-play between two important placental transcription factors and characterizes a link between structural domains and transcriptional activity of human DLX3 in human trophoblast-derived cells, therefore providing potentially important insights into DLX3- and GCM1-related gene regulatory network underlying placental cell function. Based on previously reported involvement of DLX3 and GCM1 in the pathophysiology of PE-complicated placentae in human patients^46, 54^, our *in vitro* study may also implicate novel perspectives in targeting molecular pathways contributing to maternal disease associated with PE.

## Materials and Methods

### Plasmids and reagents

PKH3-DLX3, the *CGA* promoter luciferase reporter and the *PGF* promoter luciferase reporter plasmids are described in previous studies^31, 32^. HA-tagged GCM1 was generously provided by Dr. Hungwen Chen (Institute of Biological Chemistry, Academia Sinica, Taiwan). Series of structural domains of DLX3 were constructed by polymerase chain reaction (PCR) using pKH3-DLX3 as the template and the following primer sets: DLX3^ND^ (DLX3^1-128^), forward 5′-CGGGAATTCAAATGAGCGG-3′ and reverse 5′-ATCGATTCACTTCTTGGGCTTCCCATTCAC-3′; DLX3^∆CD^ (DLX3^1-192^), forward 5′-CCGGGAATTCAAATGAGCGG and reverse 5′-ATCGATTCACACCTCCCCGTTCTTGTAGAG-3′; DLX3^∆ND^ (DLX3^125-287^), forward 5′-GAATTCAAAAGCCCAAGAAGGTCCGAAAG-3′ and reverse 5′-GGCCATCGATTCAGTACACA-3′; DLX3^CD^ (DLX3^194-287^), forward 5′-GAATTCAACCGCTGGAGCACAGTCCCAATA-3′ and reverse 5′-GGCCATCGATTCAGTACACA-3′; DLX3^HD^ (DLX3^125-194^), forward 5′-GAATTCAAAAGCCCAAGAAGGTCCGAAAG-3′ and reverse 5′-ATCGATTCACACCTCCCCGTTCTTGTAGAG-3′. To construct the TDO mutant coding sequence, the pKH3-DLX3 was used as the template and three rounds of PCR were carried out with the following primer sets: first round, forward 5′-CCGGGAATTCAAATGAGCGG-3′ and reverse 5′-AGCGGCACCTGTTCTTGTAGAGTTTCTTGA-3′; second round, forward 5′-CTACAAGAACAGGTGCCGCTGGAGCACAGT-3′ and reverse 5′-GGCCATCGATTCAGTACACA-3′; third round, forward 5′-CCGGGAATTCAAATGAGCGG-3′ and reverse 5′-GGCCATCGATTCAGTACACA-3′. *Ec*oRI and *Cl*aI restriction sites were incorporated into 5′ and 3′ primers respectively to facilitate cloning. All sequences were verified by nucleotide sequence analyses and incorporated into the pHK3 vector to generate HA-tagged DLX3 domain expressing constructs. Antibodies used in this study included: rabbit anti-DLX3 (ab66390, Abcam, Cambridge UK), goat anti-GCM1 (sc-69407X, Santa Cruz Biotechnology, Inc., Dallas, TX), mouse anti-HA (sc-7392, Santa Cruz), nonspecific rabbit IgG isotype control (ChIP Grade) (ab171870, Abcam), nonspecific goat IgG isotype control (ab37373, Abcam), HRP-conjugated VeriBlot for IP secondary antibody (ab13166, Abcam, Cambridge, United Kingdom). The proteasome inhibitor MG-132 (M7449, Sigma-Aldrich) was used with a final concentration of 10 μM in culture media.

### Cell culture

The human choriocarcinoma cell line JEG-3^71^ was purchased from American Type Culture Collection (30-2003). HTR-8/SVneo is the immortalized extravillous trophoblast cell line derived from human first trimester placenta^72^ and was generously provided by Dr. Charles Graham (Queen’s University, Ontario, Canada). JEG-3 and HTR-8/SVneo were cultured in Dulbeco’s Modified Eagle Medium (Sigma-Aldrich, St. Louis, MO) supplemented with 10% (v/v) fetal bovine serum (FBS) (Sigma-Aldrich) and RPMI 1640 Medium (Invitrogen, Carlsbad, CA) supplemented with 5% (v/v) FBS respectively. Media were also supplemented with 100 units/mL penicillin G, and 100 μg/ml streptomycin (Invitrogen). Cells were incubated in a humidified 5% CO_2_ atmosphere at 37 °C.

### Transient transfection and luciferase assays

Transient transfection was performed in dishes of cells with 60~80% confluency using the polyethylenimine reagent (Polysciences, Inc. Warrington, PA) per manufacturer’s protocol. Corresponding empty vectors of expression plasmids were used as substitutions to make equal amounts of total DNA transfected across dishes. Eight hours after transfection, cells were washed with phosphate-buffered saline (PBS), and supplemented with fresh serum-containing media. Cells incubated for 48 h were collected and lysed in luciferase buffer (Promega, Madison, WI). Luciferase activity was quantified using the LB9501 luminometer (Berthold Technologies, Germany) and standardized by total cell protein content as determined by Bradford assay.

### Co-immunoprecipitation (IP)

JEG-3 cells were transfected with pHA-GCM1 for 48 h followed by MG-132 treatment for 6 h. Cells were then collected in lysis buffer (1% Nonidet P-40, 150 mM NaCl, 50 mM Tris-HCl, pH 7.5) freshly supplemented with complete protease inhibitor cocktail (Roche, Basel, Switzerland) by rotation at 4 °C for 1 hr. For each IP, 500 μg whole cell lysate was incubated with 5 µg primary antibody at 4 °C for 1 h with rotation. Protein A Dynabeads (Invitrogen, Carlsbad, CA) were then added into samples and incubated at 4 °C with rotation for an additional hour. IP- antibody-beads were collected using a magnet, washed twice with lysis buffer and once with TPBS (0.01% Tween®−20, pH 7.4), and eluted in 50 μl 2 × Laemmli buffer at 95 °C for 5 min. IPs were subsequently resolved by SDS-PAGE and subjected to Western blot analyses.

### Western blot

Cell lysates or IPs were resolved on SDS-polyacrylamide gels and transferred to polyvinylidene difluoride (PVDF) membrane (Bio-Rad, Hercules, CA). Membrane was blocked with 5% milk-TBST and probed with the primary antibody (1:1,000 dilution), HRP-conjugated secondary antibody (1:3,000 dilution), and ECL reagents (Bio-Rad).

### Mammalian one-hybrid assay

GCM1 cDNA was obtained by PCR using the pHA-GCM1 plasmid as the template and the following primers: forward 5′-AATCCATGGAACCTGACGACTTTGA-3′ and reverse 5′-TCTAGAGTCATCTCAAAGGACACAGGTT-3′. *Eco*RI and *Xba* were added to the 5′ and 3′ end of GCM1 cDNA sequence respectively to facilitate cloning. The sequence was verified by nucleotide-sequencing analysis. To construct the GAL4 DBD-GCM1 fusion protein, GCM1 cDNA was incorporated in frame and downstream of the GAL4 DBD (residues 1-147) of the pSG424 plasmid^73^. JEG-3 or HTR-8/SVneo cells were seeded in 35 mm wells at a 60~70% confluency before transfection. Cells were transfected with 0.5 μg pSG424-GCM1 (Gal4 DBD-GCM1), 0.5 μg pGL4.31 luciferase reporter (Promega, Madison, WI) containing five-repeats of the GAL4 binding site (5 × GAL4 binding sites) adjacent to the minimal TATA box of major late adenovirus promoter, and 0.5 μg or 1 μg pKH3-DLX3 or DLX3 domain expression plasmids for 48 h followed by MG-132 treatment for 6 h. Cells were then collected for luciferase assays.

### Statistics

Data are presented as mean ± standard error (SEM) from an individual representative of three independent experiments. One-way analysis of variance (ANOVA) followed by Student’s unpaired *t* tests were used to compare the means between DLX3^WT^ and DLX3 functional domains, and DLX3^WT^ and DLX3^TDO^. Two-way ANOVA followed by the post-hoc Bonferroni tests were used multiple comparisons were performed between GCM1 and DLX3^WT^, GCM1 and DLX3 functional domains and GCM1 and DLX3^TDO^ in luciferase reporter and mammalian one hybrid assays. A *P* value of less than 0.05 was considered to be statistically significant.

## Electronic supplementary material

Supplemental Data
