# Supplementary material for: DLX3 interacts with GCM1 and inhibits its transactivation-stimulating activity in a homeodomain-dependent manner in human trophoblast-derived cells
Source: Sci Rep. 2017 May 17;7:2009. doi: 10.1038/s41598-017-02120-5 (PMC5435702; doi:10.1038/s41598-017-02120-5)

DLX3 interacts with GCM1 and inhibits its transactivation-stimulating activity in a homeodomain-dependent manner in human trophoblast-derived cells

Sha Li and Mark S. Roberson

Department of Biomedical Sciences, College of Veterinary Medicine,

Cornell University, Ithaca, NY

Supplementary Data

Supplementary Figure Legends

Supplementary Figure 1. Uncropped HA western blots for HA-tagged Dlx3 and β-actin lane loading controls with molecular size standards included for Figure 1B.

Supplementary Figure 2. Uncropped HA western blots for HA-tagged Dlx3 structure function mutants following GCM1 immunoprecipitation with molecular size standards and IgG controls included for Figure 3 A-F.

Supplementary Figure 3. Uncropped HA western blots for HA-tagged $Dlx3^{WT}$, HA-tagged $Dlx3^{TDO}$ mutant and HA-tagged GCM1 with molecular size standards included for Figure 7.

Li and Roberson, 2017
Supplementary Figure 1

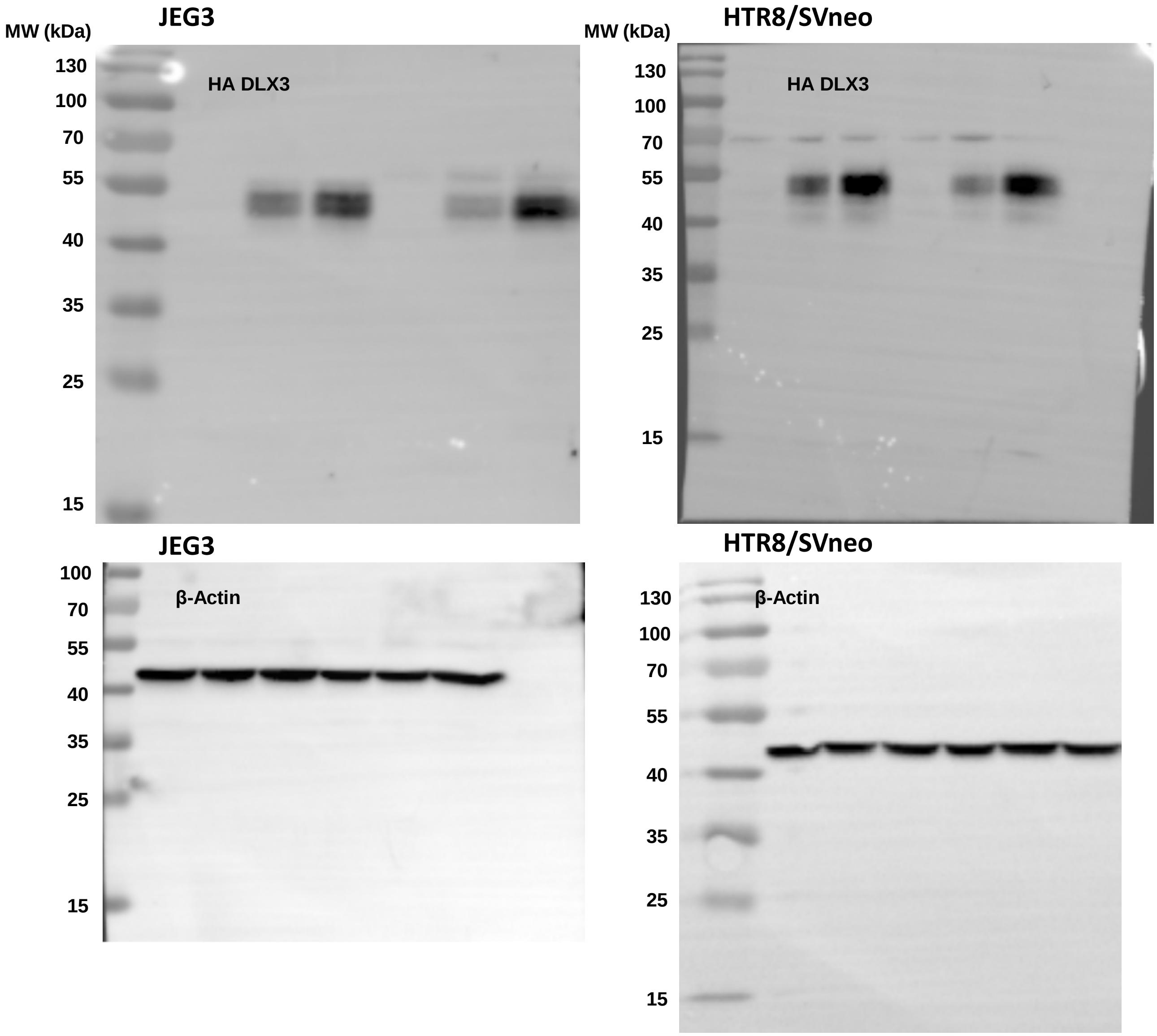

Li and Roberson, 2017
Supplementary Figure 2

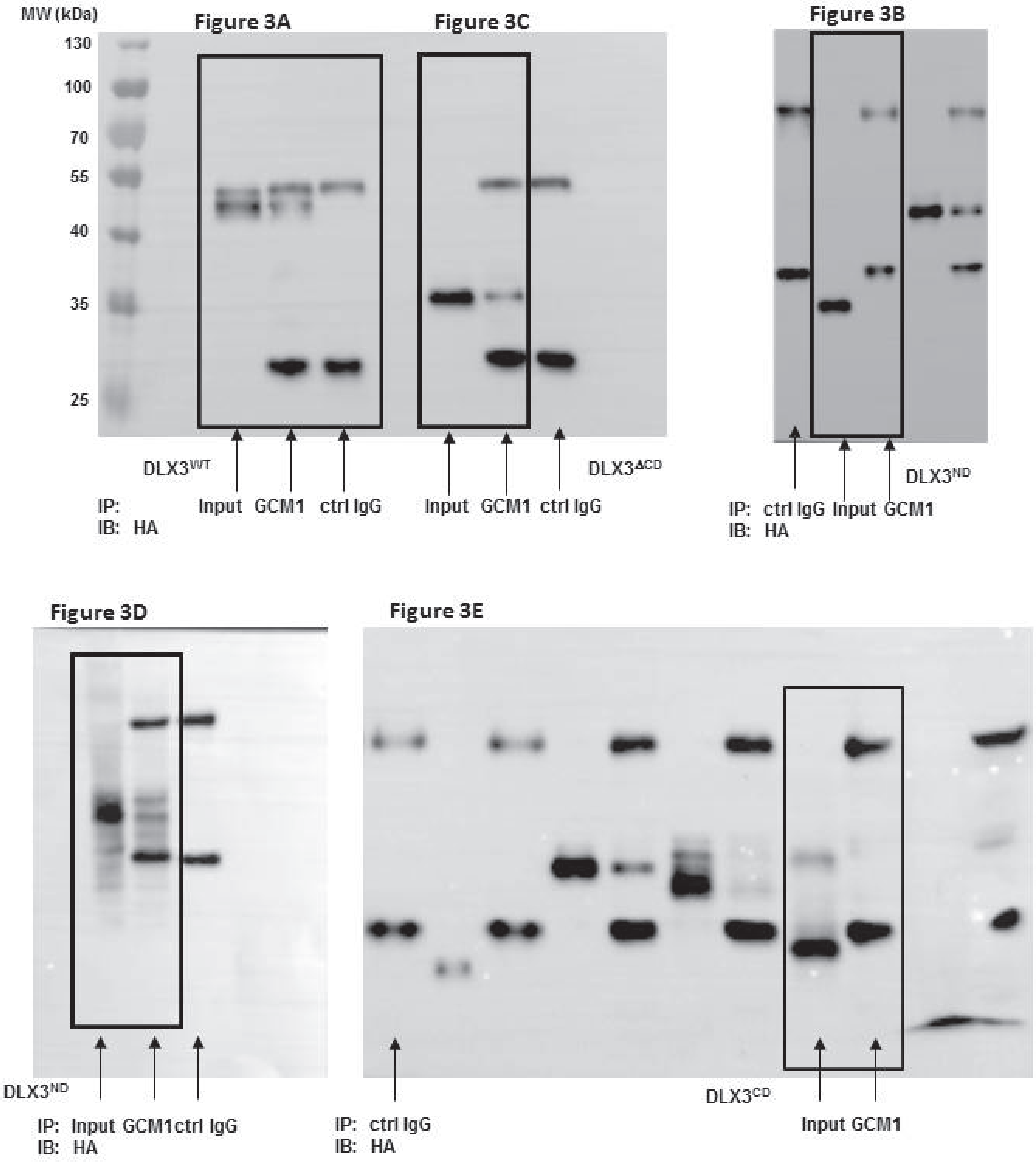

Li and Roberson, 2017

Supplementary Figure 2, Continued

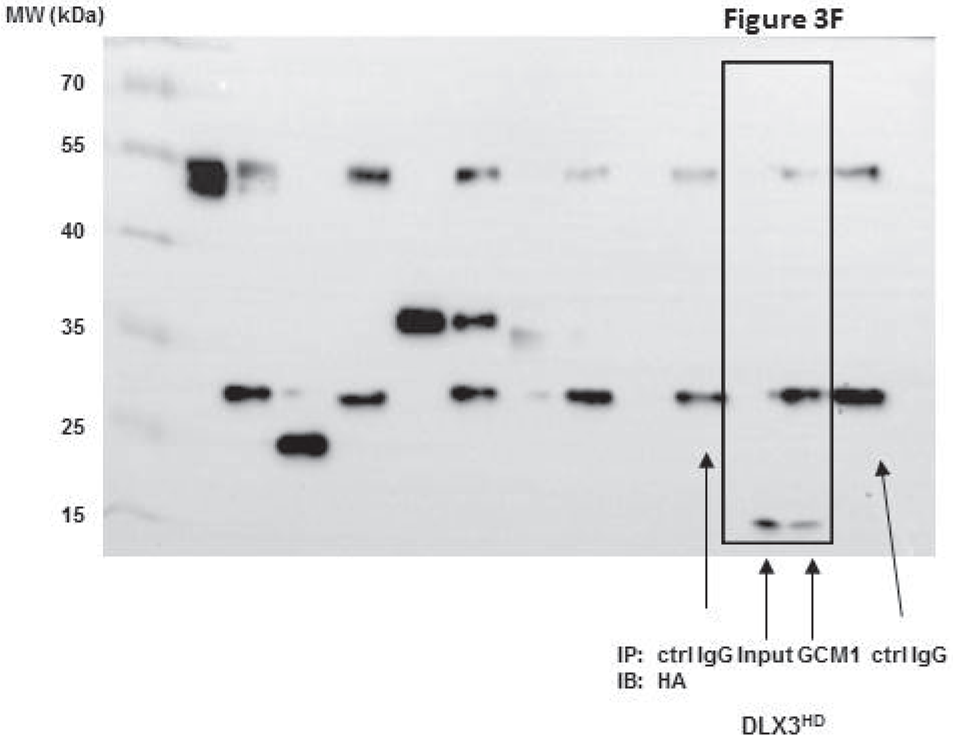

Li and Roberson, 2017
Supplementary Figure 3

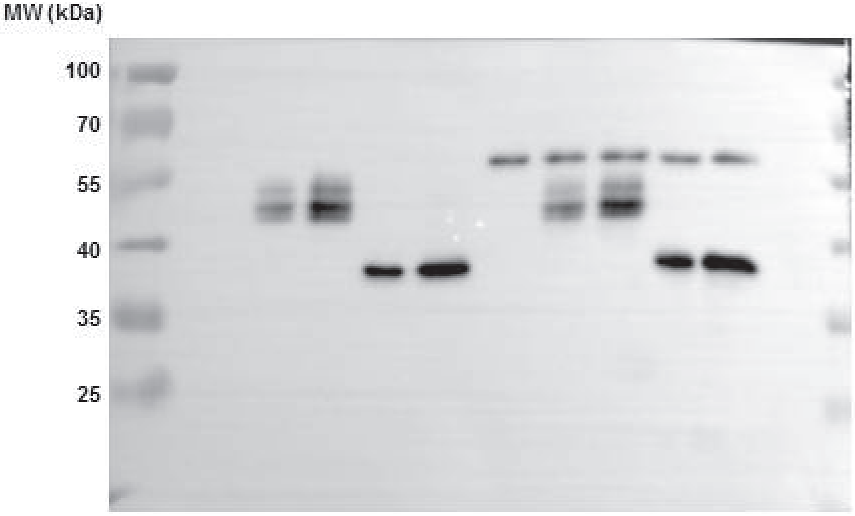

Supplement: Supplementary file 1 — Supplemental Data [file 41598_2017_2120_MOESM1_ESM.pdf]
